# Results-based aid with lasting effects: sustainability in the Salud Mesoamérica Initiative

**DOI:** 10.1186/s12992-018-0418-x

**Published:** 2018-10-16

**Authors:** Charbel El Bcheraoui, Aruna M Kamath, Emily Dansereau, Erin B Palmisano, Alexandra Schaefer, Bernardo Hernandez, Ali H Mokdad

**Affiliations:** 1 Institute for Health Metrics and Evaluation, University of Washington, 2301 5th Avenue, Suite 600, Seattle, WA 98121 USA; 2 Department of Anesthesiology, University of Washington, Seattle Children’s Hospital, Seattle, WA USA

**Keywords:** Salud Mesoamérica Initiative (SMI), Maternal and child health, Results-based aid, Central America, Dynamic Sustainability Framework (DSF), Sustainability, Implementation science

## Abstract

**Background:**

The Salud Mesoamérica Initiative is a public-private partnership aimed at reducing maternal and child morbidity and mortality for the poorest populations in Central America and the southernmost state of Mexico. Currently at the midpoint of implementation and with external funding expected to phase out by 2020, SMI’s sustainability warrants evaluation. In this study, we examine if the major SMI components fit into the Dynamic Sustainability Framework to predict whether SMI benefits could be sustainable beyond the external funding and to identify threats to sustainability.

**Methods:**

Through the 2016 Salud Mesoamérica Initiative Process Evaluation, we applied qualitative methods including document review, key informant interviews, focus group discussions, and a social network analysis to address our objective.

**Results:**

SMI’s design continuously evolves and aligns with national needs and objectives. Partnerships, the regional approach, and the results-based aid model create a culture that prioritizes health care. SMI’s sector-wide approach and knowledge-sharing framework strengthen health systems. Evidence-based practice promotes policy dialogue and scale-up of interventions.

**Conclusion:**

Most SMI elements fit within the Dynamic Sustainability Framework, suggesting a likelihood of sustainability after external funding ceases, and subsequent application of lessons learned by the global community. This includes a flexible design, partnerships and a culture of prioritizing healthcare, health systems strengthening mechanisms, policy changes, and scale-ups of interventions. However, threats to sustainability, including possible transient culture of prioritizing health care, dissipation of reputational risk and financial partnerships, and personnel turnover, need to be addressed.

**Electronic supplementary material:**

The online version of this article (10.1186/s12992-018-0418-x) contains supplementary material, which is available to authorized users.

## Background

Implementation of public health programs has traditionally resulted in short-term gains but less long-lasting impact, particularly in low-income countries with barriers in health systems, professional regulation, and evidence-based interventions [1, 2]. During the 1970s and 1980s, researchers evaluated the work abroad of large donors, such as the US Agency for International Development, and non-governmental organizations, through which contextual factors and project characteristics that influenced sustainability were identified [3–5]. Scholars continued, into the 1990s, to pose the question of the post-donor period, highlighting the judicious use of limited resources, and further conceptualizing sustainability [6, 7]. Since 2005, sustainability has gained more attention under larger movements, such as the Organisation for Economic Co-operation and Development’s Paris Declaration of Aid Effectiveness [8], the World Health Assembly’s resolution for sustainable health financing [9], and the World Health Organization’s call for health systems strengthening [10], a shift away from the Millennium Development Goals era of disease-specific investments, targeting HIV/AIDS, tuberculosis and malaria, among others [11]. Limited financial growth of development assistance globally, with more focus on disease-specific aid rather than sector-wide approaches [12], provides an opportunity to develop novel funding methods, improved donor-government and regional partnerships, robust health systems, and sustainable initiatives suitable for replication.

The Salud Mesoamérica Initiative (SMI) is a public-private partnership dedicated to improving maternal and child health among the most underserved communities in seven Central American countries and the state of Chiapas in Mexico. Initiated in 2011, SMI is a three-operation initiative, with respective goals to increase supply, improve service, and generate demand for health services. SMI focuses on five public health domains: child health, vaccines, family planning, antenatal care, and peripartum care. Within these domains, specific performance indicators, such as health facilities must have permanent supplies of five types of modern family planning methods or antenatal care by a qualified personnel must start before 12 weeks of gestation, are measured at the end of each operation. During the first operation, almost all targets set for SMI have been met, or exceeded, by participating countries. For example, the percent of health facilities with cold chain managed according to norms increased from 28.6 to 88.9% in Nicaragua, the percent of health facilities with permanent availability of of supplies and equipment necessary for pediatric, vaccination, and nutrition care increased from 11.8 to 84.2% in Panama, and health facilities with permanent availability of modern family planning supplies according to norms increased from 19.0 to 92.2% in El Salvador [13–20]. With SMI having been successful thus far in meeting its targets per the first operation findings, the sustainability of the initiative deserves investigation to ensure the gains acquired will continue once external funding ceases.

For the purpose of this analysis, we use the Organisation for Economic Co-operation and Development definition of sustainability, “the continuation of benefits from a development intervention after major development assistance has been completed, with the probability of continued long-term benefits” [21]. Results-based aid (RBA) is defined as a financial assistance model involving a contract between a partnering development donor and the government recipient with defined incentives, measurable results, and aid disbursement based upon performance or predetermined targets [22].

Few conceptual models of sustainability exist, and those that do often focus on health programs in developed countries [2, 23–28]. Of the limited sustainability models with a global health application in low-resource settings [6, 7, 29–31], we chose the Dynamic Sustainability Framework (DSF) to evaluate SMI’s potential for sustainability as it emphasizes sustainability as a dynamic rather than a linear or static process, one that recognizes change occuring since the initial intervention, and the need for continuous improvement and ongoing evaluation [32, 33]. Compared to earlier global health frameworks that define sustainability and conceptualize the influential factors of project design, organizational setting and environmental setting [6, 7, 29, 31], the DSF expands upon this by noting adaptive and contextually sensitive interactions. While Gruen et al. also emphasizes a dynamic process, this proposed framework has not been applied to real-world examples [30]. The DSF has been cited in a systematic review of health programs in Africa and is thus a better choice for this analysis [32].

The DSF states that a sustainable intervention is one that presents the following seven tenets: 1) intervention is not optimized before implementation; 2) intervention continually improves and enables ongoing learning; 3) ongoing feedback on interventions uses relevant measures of progress; 4) ongoing stakeholder involvement occurs; 5) fit between program and setting exists; 6) organizational learning is a core value; and 7) voltage drop is not inevitable, which means that an intervention does not necessarily yield lower benefits to the recipients of the intervention when implemented in the real world. The concept of voltage drop has often been accepted as many interventions fail to achieve the original outcomes observed which might be due to reduced fidelity to the intervention when implemented by community-based organizations outside of the academic setting, or to a lack of guidance in adapting the intervention to the target population [34].

In this study, we explored whether the SMI design and components align with the DSF as a means of forecasting potential long-term benefits. In doing so, we aimed to predict whether the gains of SMI documented so far are likely to be sustained when external funding ceases, to identify barriers to sustainability that may need to be addressed, and to discuss lessons learned about sustainability from this initiative that may be replicable in other regions of the world.

## Methods

From May to October 2016, qualitative data collection was conducted for the Salud Mesoamérica Initiative Process Evaluation. Qualitative methods consisted of document review, key informant interviews (KII), a social network analysis, and focus group discussions (FGD). By triangulating these different methods, we sought to gain a comprehensive assessment of SMI with input from stakeholders at the regional, national, and local levels (Additional file 1).

### Document review

We reviewed the initiative’s theory of change (TOC), operational plans, initiative proposals, annual and quarterly progress reports, national health plans, intervention plans developed for specific health areas, local official reports, needs assessment reports, and monitoring and evaluation documents. Here, we emphasize that the TOC is not used as a framework to guide our analysis but as material analyzed within the DSF first and second tenets.

### Key informant interviews

#### Topic guide development

We generated topic guides for KIIs based on the document review, stakeholder exchanges, and fact-finding trips. Topic guides concentrated on issues pertaining to SMI planning, design, implementation, theory of change, efficiency, and lessons learned, as well as stakeholders’ decisions, resource allocation, and effectiveness (Additional files 2 and 3).

#### Sample selection

To be eligible, key informants (KIs) selected must have been involved in one or more of the following: design and funding of SMI in general; design, planning, and implementation of the SMI operation for Chiapas in Mexico; or working at the ministry of health in an SMI country. KIs fell into two main groups: decision-makers and programmatic actors. The decision-maker group was composed of SMI partner organizations, including SMI funders (global key informants), Inter-American Development Bank (IDB), technical assistance partners, ministries of health from multiple SMI countries (national key informants), and the Chiapas ministry of health (local key informants). The programmatic actor group consisted of health care providers and managers of health care facilities in Chiapas.

### Social network analysis

#### Survey tool design

We used the PARTNER (Program to Analyze, Record and Track Networks to Enhance Relationships) tool, designed by the University of Colorado, to demonstrate how members are connected, assess the degree of collaboration and engagement among stakeholders over time, evaluate the level of trust in the initiative network, and understand SMI objectives and outcomes. KIs representing the organizational stakeholders of SMI were asked to respond to questions measuring their perception of partner organizations, and about SMI in general, and within the state of Chiapas in Mexico. They also responded to multiple-choice questions regarding their views on SMI’s objectives, success, and aspects of collaborative work that propel the success it has achieved so far.

### Focus group discussions

#### Topic guide development

To gain a regional-level perspective of the SMI design and implementation from countries’ perspectives, a topic guide was created for representatives of the Ministries of Health from SMI countries involved in SMI implementation in their respective countries (Additional file 4).

#### Sample selection

Ministry of health representatives from Honduras, Guatemala, Costa Rica, Belize, El Salvador, and Panama were present at the Latin American and Caribbean Conference 2016, and concurrently served as the nine participants sampled for the focus group discussion (FGD).

### Data analysis

All interviews from KII and FGD were transcribed verbatim from the audio recordings, and if needed, professionally translated to English. Next, four researchers with educational backgrounds in health metrics, public health program evaluation, or social sciences manually coded and analyzed interview content through recursive abstraction. This thematic analysis followed a deductive approach based on the DSF tenets. The generated codes were used to identify general themes that matched the DSF dimensions. Data collected through the PARTNER tool were analyzed for connections between respondents to visualize the centrality of each organization and the number of organizations it is connected to. This allowed us to verify whether any outliers existed in the partnership.

### Ethical considerations

The evaluation received Institutional Review Board exemption as a non-human-subject research determination from the University of Washington, and verbal consent was obtained from all study participants prior to interviews and data collection (Additional file 5). For the KII and FGD, participants were informed that responses would remain anonymous and confidential. Interviews ranged in duration from 60 to 90 min, and were conducted in the interviewee’s native language with an interpreter if necessary, and primarily in an in-person format. For the social network analysis, a de-identified questionnaire was administered on paper or online based on the KI’s preference. All questions were related to the Salud Mesoamérica Initiative, and not to the KIs.

## Results

Between May and October of 2016, we interviewed 113 key informants and one focus group on the sustainability, relevance, effectiveness, and efficiency of SMI. Six representatives from the donors, the Instituto de Salud del Estado de Chiapas (ISECH), and the Secretaría de Salud (SSA) were not available for interviews, and a couple of health care providers declined participation without providing a reason. Twenty out of 23 organizations, in SMI Chiapas participated in the social network survey. One donor, one ISECH department, and one SSA department did not respond to the partnership survey. Four themes on sustainability emerged relating to the tenets of the DSF (Table 1), with corresponding DSF tenets noted in brackets.

**Table 1:** DSF tenets, SMI results, and threats to sustainability

DSF tenets	SMI results	Threats to sustainability
Not optimized before implementation (Tenet 1)	✓ Evolving design	
Continually improves and ongoing learning (Tenet 2) Ongoing feedback (Tenet 3)	✓ Evidence-based practices ✓ Sector-wide approach (quality improvements, capacity building, health information systems, knowledge-sharing)	
Voltage drop not inevitable (Tenet 4)	✓ Culture of prioritizing healthcare ✓ Policy dialogue ✓ Scale-up of interventions	✓ Possible transient culture of prioritizing healthcare
Fit between program and setting (Tenet 5)	✓ Aligns with national needs ✓ Health systems strengthening	
Organizational learning (Tenet 6)	✓ Problem-solving capacity	✓ Personnel turnover
Ongoing stakeholder involvement (Tenet 7)	✓ Partnerships, including regional approach and RBA model	✓ Dissipation of reputational risk and donor-government financial partnerships

### Theme 1: the intervention design evolved and aligned with national needs over time [tenets 1, 5]

**Fig. 1:**
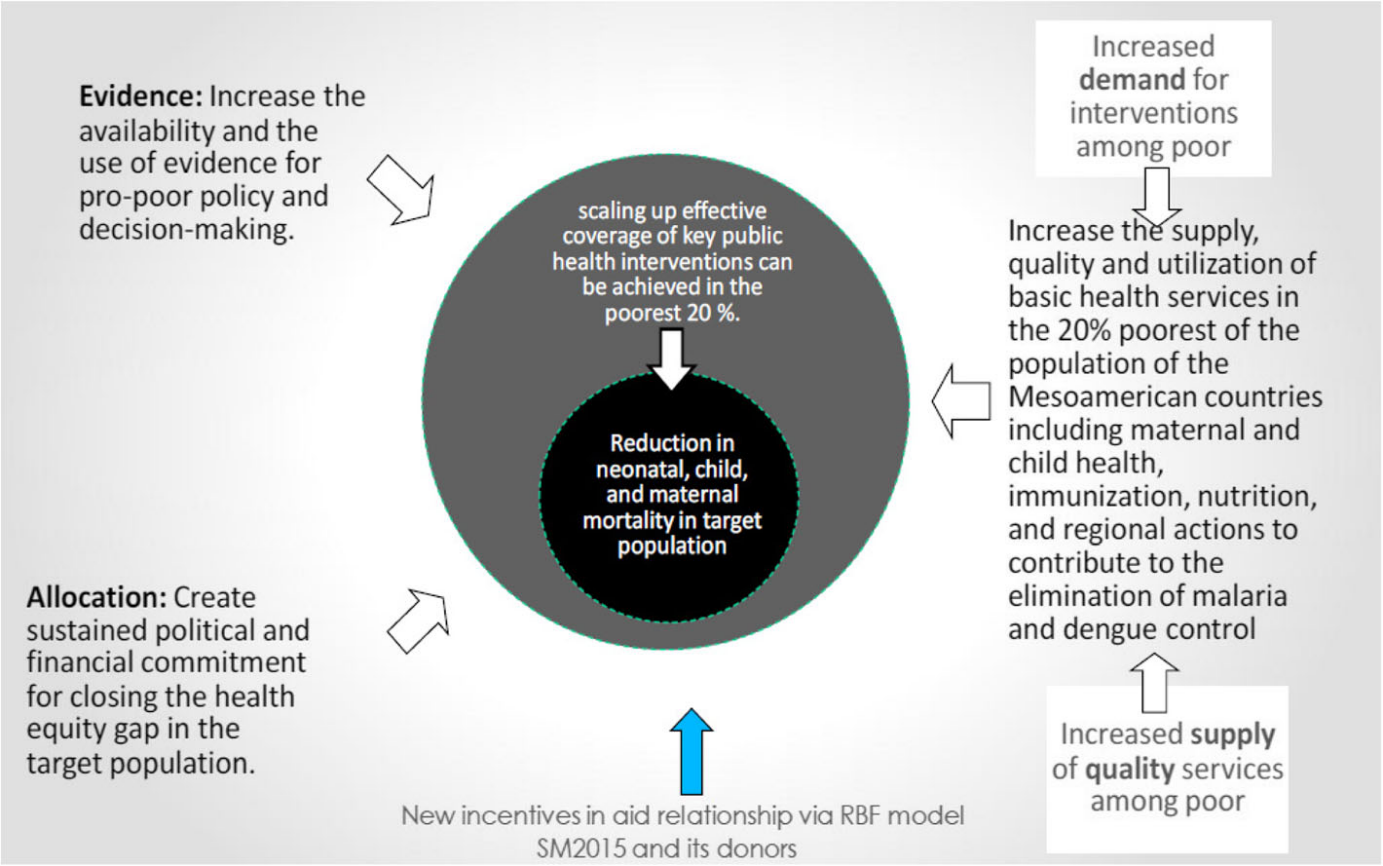
Initial Theory of Change of the Salud Mesoamerica Initiative. Source: Interamerican Development Bank, 2011. Monitoring and Evaluation Plan: Salud Mesoamerica 2015 Initiative

**Fig. 2:**
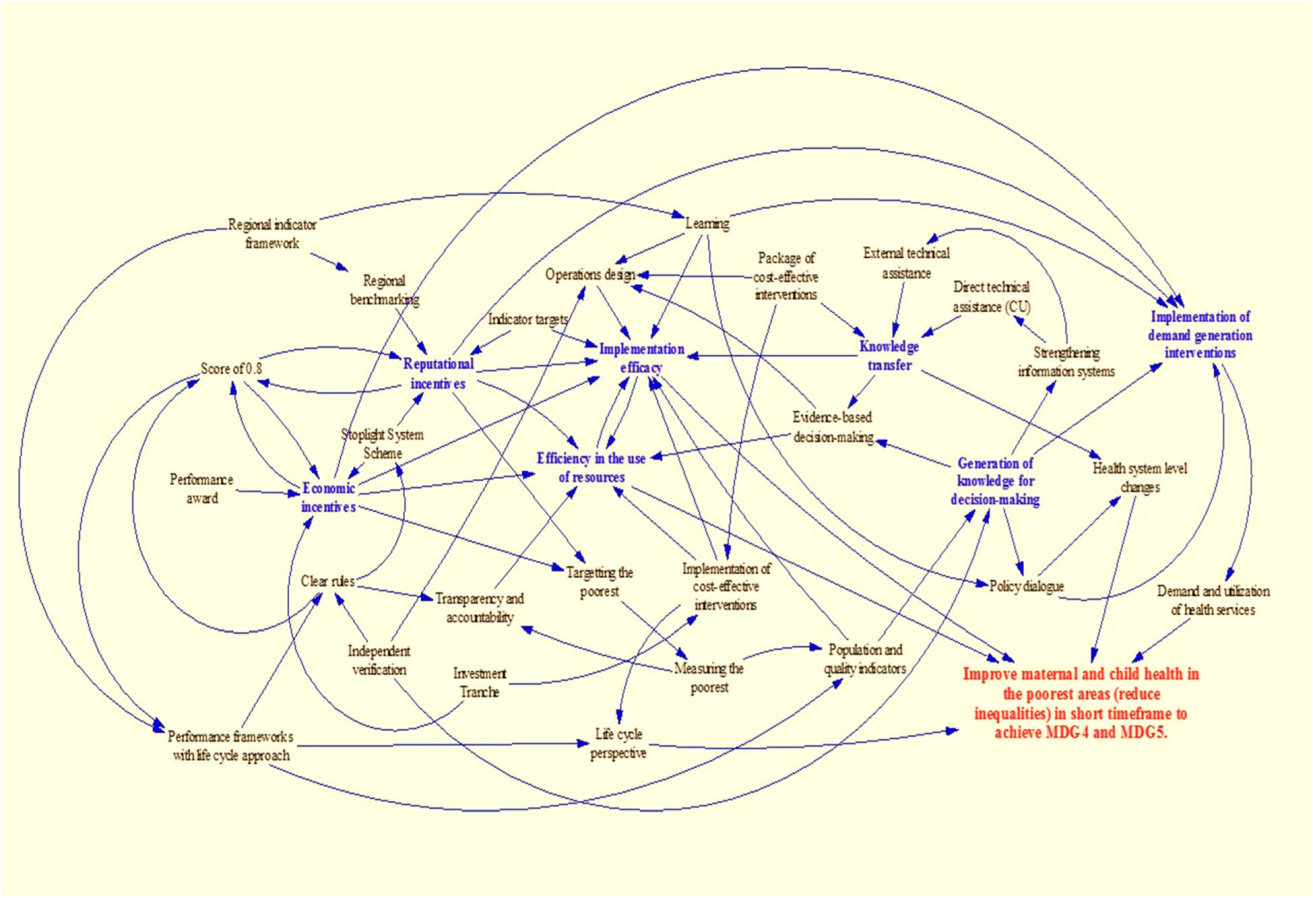
Causal-loop model of the Salud Mesoamerica Initiative. Source: Interamerican Development Bank, Salud Mesoamerica Initiative, internal communication

The overarching design of SMI is steered by a flexible, evolving theory of change (TOC) as observed in the reviewed documents. Over time, this TOC moved away from a simple framework to incorporate supply, demand, allocation, and evidence pathways (Fig. 1) [35] and eventually toward a more comprehensive, causal-loop model (Fig. 2) [36] geared toward the long-term goal of reducing maternal and child mortality and morbidity. The updated TOC captures the conceptual questions of what, why, and how components of the project work, with the fluidity to add evidence, integrate changing practices, and constantly gather input from context, knowledge, and policy assessments.

*“One big thing about the theory of change of SMI is that you make these changes through a learning environment.”* – *Donor*

*“So what I’m saying, it should have a degree of adaptability and flexibility built into the program. … We want to know how to do better.”* – *IDB*

With this reflective learning environment, the TOC evolves, and more importantly mirrors the continuous development of SMI’s design and aligns with country priorities. According to ministry of health informants, SMI’s long-term goal not only matched, but catalyzed forward, the existing national health priorities.

*“I believe there are two elements where the initiative has aligned itself very well with the country initiatives. And it aligned to improve – to empower the country initiatives. And the two elements are in the decentralization process, as we call it in Honduras.… This has been an initiative of the Honduras health secretariat. And SMI abided by that country decision. And with their strategy and co-financing, they’ve been able to empower that country initiative. The other one is the one that is an effort being carried out by the country for many years. It’s the reduction of maternal and child mortality. And the initiative is strongly aiming towards that goal to improve access of women and children to health services, as well as improving the response capabilities of the services to the mother and child.” – Honduras*

SMI performance indicators, such as the ones around vaccine coverage, family planning, and pregnancy management, were derived from the health standards of each country, and the SMI long-term goal of reducing maternal and child mortality and morbidity was a shared objective of national health plans.

### Theme 2: the partnerships, regional approach, and results-based aid created a culture that prioritizes healthcare [tenets 4, 7]

**Fig. 3:**
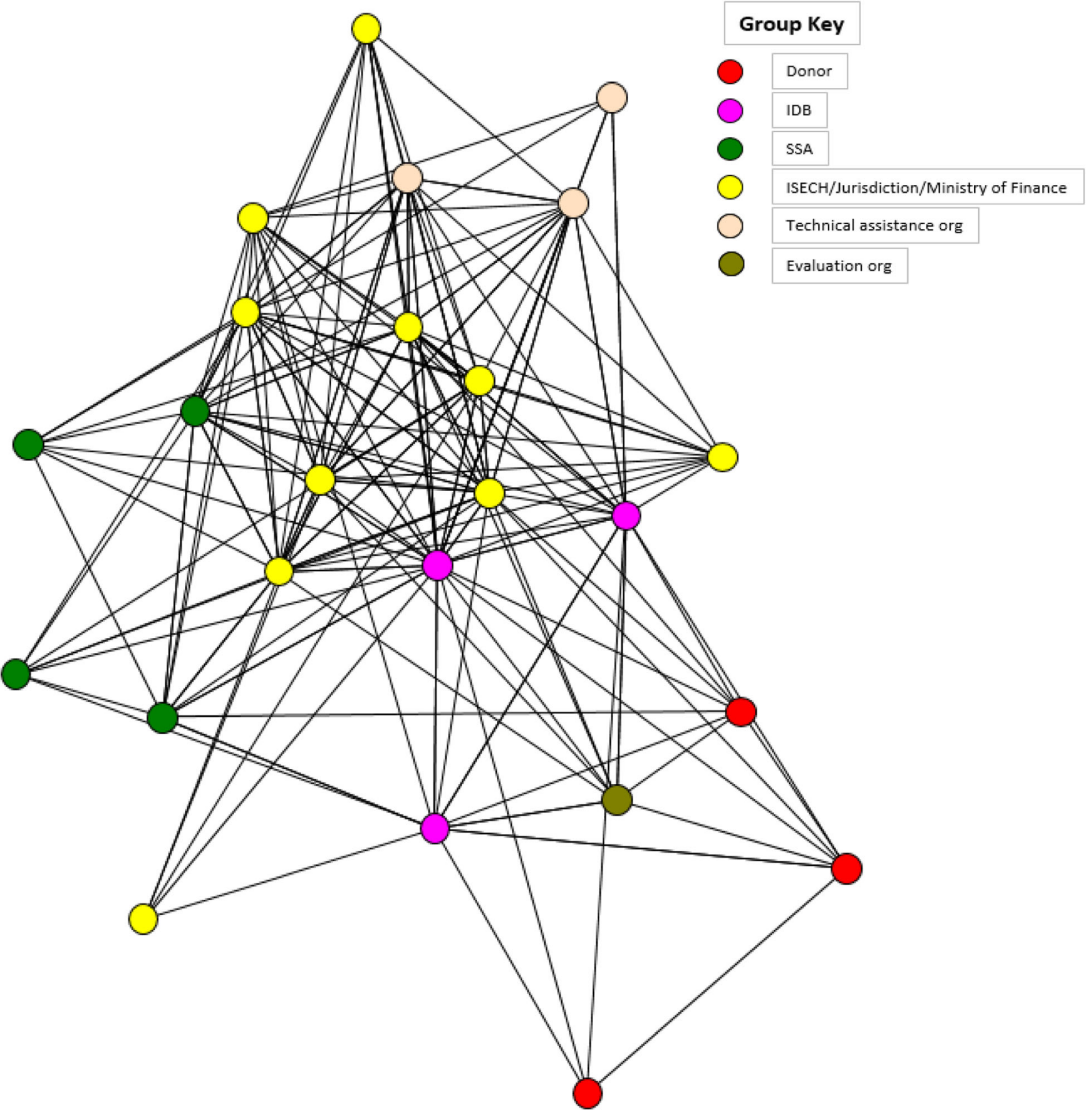
Partnership network of the Salud Mesoamerica Initiative

SMI is an immense undertaking, with partnerships ranging from external funders, namely the Bill & Melinda Gates Foundation, the Carlos Slim Foundation, and the Spanish Agency for International Development Cooperation; to an independent facilitator, the Inter-American Development Bank; to each country’s Ministry of Health and subsequent state departments of health; to the local health care workers; and to technical assistance partners. Based on KIIs, FGDs, and the social network analysis, these diverse stakeholders not only contributed within their individual domain, but also demonstrated the ability to work together on all appropriate levels and stages of the project in a collaborative and coordinated effort. Figure 3 from the social network analysis shows a high degree of interconnectedness among all stakeholders, namely donors, IDB, SSA, ISECH, jurisdiction, ministry of finance, technical assistance, and evaluation organizations. For instance, as seen in Fig. 3, no member was isolated from the network: all members collaborated or connected with at least four other partners, and with the strongest ties in the center of the network at the state level, ISECH and jurisdiction [37]. .Beginning with the inception of SMI, input and engagement from all was a priority as reported by KIs and shown in the reviewed documents. For example, the earliest stage involved outlining the key interventions of maternal and child health known as the Master Plans. This step demanded active participation by donors and in-country representatives alike through regional workshops and joint meetings.

*“So we actually started to dialog with the countries with any people in the ministries and with the minister of finance … to understand the type of mechanism where we are going to promote.… And once they say okay, we think it’s a good idea let’s start work*.*” – IDB*

Over time, partnerships grew stronger and remained essential to the survival of SMI, leading to local leadership, ownership, and higher levels of trust. In the case of Mexico, SSA and ISECH, representing the federal and state health departments, respectively, recognized the need to work synergistically in order to achieve a shared vision after the country failed to meet the target indicators during the first phase.

*“That was a big change and the fact that they failed was also, I think, the part of the drive so that they decided to change the way they were approaching things and, of course, there was also a federal response to be closer, to work more closely with the state of Chiapas and supervision actions. I think everyone had a role to play, but I think the change at the state level, the jurisdictions, was the key to this.” – SSA*

Per KIs, this synergistic partnership has reportedly led to building ministry of health ownership and an executive role by ISECH and the Chiapas project coordinating unit, in terms of the planning, monitoring, target attainment, and operation of the project locally. The social network analysis survey of more than 20 stakeholders in Chiapas also showed trusting partnerships, with the project coordinating unit within ISECH and Dirección de Atención Médica at ISECH rated as the most trusted in the network, alongside IDB, and the Bill & Melinda Gates Foundation. For the same reason, ISECH reportedly felt the need to more actively engage health workers from the field who knew the situation better on the ground, which promoted inclusiveness and community ownership. Key informants note their observation that community-level actors gained a sense of accountability and ownership through process improvements, output goals, and quality check activities, such as checking input registries on a daily basis. However, despite the strong partnership at almost all levels, health workers from some of the most marginalized areas reported feeling isolated.

The regional approach and RBA model distinguish SMI from other global health initiatives, offering specific benefits. Rather than focusing on one country, SMI implemented maternal and child health interventions in eight countries within the same geographical region. In doing so, this regional approach unexpectedly introduced a reputational risk and desire to reach indicator targets. Per KI sources, this peer pressure environment served as an important driving factor to meeting SMI indicators because a country’s reputation was at stake.

*“Now, with this one, it offers to share the risk of the donor with the beneficiary because I don’t just have the fear of losing but also the fear of not winning, of not earning. So it’s an extra encouragement for the country to be more efficient, to make greater efforts to seek the results. I believe that this is an additional stimulus. And the fact that it’s also with other countries: it adds an additional pressure on top of that additional pressure. Because we don’t want to end up looking bad. So we’re always aware and pending*.*”* – Honduras

A joint meeting by the Ministries of Health of Central America, called Consejo de Ministros de Salud de Centroamérica (COMISCA), which is held biannually, reportedly instilled this regional competition further by publicly presenting countries based on their SMI performance. The regional approach also ignited inter-country learning, through which ministry of health stakeholders compared and improved the efficiency of interventions and best practices, technical assistance, and economies of scale for supply procurement.

From a financial perspective, the RBA model reportedly brought accountability and ownership to the SMI endeavor. Unlike other performance-based aid models traditionally used in global health initiatives, RBA emphasizes a country-led investment. RBA requires co-financing, in which governments and donors provide equal funding at baseline. SMI is a three-operation initiative, concentrating on inputs for the health system in the completed first operation, and focusing on service delivery and demand generation in the current and upcoming operations. Decided upon mutually by governments and donors, a set of payment indicators for each operation serve as targets for reducing maternal and child health morbidity and mortality. If 80% of indicator targets are met, a performance tranche, or portion of the baseline funding, is awarded to be reinvested in the health system at the discretion of the government, and the country advances on to the next phase of the initiative. These financial incentives of the RBA model, along with driving factors of the regional approach, ensured an ongoing heightened commitment by country stakeholders.

Collectively, partnerships, the regional approach, and the RBA model of SMI create a culture that prioritizes healthcare and permeates from a regional to local level according to KIs.

*“The quality of healthcare has to be embedded in the culture, and it is not all about having certain resources or medical knowledge, but it is also about committing to the service and having the right attitude to work as a team with others*.*”* – *SSA*

Stronger partnerships promote a culture of collaboration, inclusiveness, and engagement. The regional approach creates a reputational risk and promotes inter-country learning around quality and efficiency. RBA establishes a culture of ownership and accountability, specifically in the case of Chiapas, moving away from a culture of unmonitored aid.

*“Within ISECH, who have begun to see SMI as their own project rather than one imposed by external forces.” – ISECH*

Such positive attitudes have become the new norm and expectation in the health care sector of these countries.

Some KIs express the possibility that this culture of prioritizing health care may be fleeting.

*“It’s not something that will be guaranteed because we know that sometimes it depends on people if this is not institutionalized.” – SSA*

At this snapshot in time, all stakeholders show ongoing involvement in the SMI project. However, this current participation does not translate into predicting ongoing involvement in future phases of the project. When funding ceases, by default, players that are not in-country stakeholders must exit. Reputational risk of the regional approach and donor-government financial partnerships of the RBA model might dissipate. Consequently, certain aspects of the prioritization of health care might decline. Without the drive to meet payment indicators, the donor-government partnership of the RBA model and the healthy competition of the regional approach might fade. However, aspects such as inter-country learning and ownership will likely persist, according to other KIs. Some stakeholders call for stronger social auditing to safeguard accountability of governments to the populations affected.

### Theme 3: the sector-wide approach and knowledge-sharing strengthened the health system [tenets 2, 3, 5, 6]

Rather than a patchwork effort to meet target goals, SMI implemented a sector-wide approach to strengthen the operational system of each country. Quality improvement, capacity building, revamping health information systems, and knowledge-sharing are at the core of this sector-wide approach.

Quality improvement initiatives focused on process improvements and management practices to improve efficiency and quality of care. First, in terms of process improvements, the external technical assistance partner, Management Sciences for Health (MSH), funded by and under the guidance of SMI, worked jointly with a mirror team from ISECH at a sample of health facilities to investigate gaps in quality of care and to create standards for quality improvement. For example, the ISECH mirror team learned implementation skills for service delivery and cultural adaptations for family planning and obstetric and neonatal care from MSH at a sample clinic, and then independently reproduced quality improvement goals in other SMI areas. Another quality improvement example in Chiapas is the community engagement of midwives to address cultural sensitivity around pregnancy, as well as training midwives to detect high-risk pregnancies and refer these patients to clinics.

Second, in terms of management practices, health care workers report improved supervision, communication, trainings, and standardization of work processes and recordkeeping.

*“In the past we didn’t have much training, and now we even get two or three people from our nurse staff who go to receive training in San Cristobal and then teach their coworkers what they learned there. That way everyone is informed and we can keep training our staff.” – Doctor*

Guidelines, such as the revised cold chain protocol for vaccines and new obstetric emergency alert systems, have been executed and have demonstrated valuable outcomes.

Capacity building, with an emphasis on human resources and physical supplies, has met major challenges but showed improvement with SMI interventions. Per health care workers and KIs, obstacles noted are workforce shortages and frequent turnover of personnel and administration, as well as a stockouts of various equipment and medications in health facilities.

*“When there is an emergency, and we need to transfer the patient, my only doctor has to take that woman to a second level hospital, leaving no one here in case someone needs to assist a delivery. We need more human resources.” – Health facility director*

In response, SMI in Chiapas provided quality training for health care personnel on topics such as maternal home care and the Essential Obstetric and Newborn Care strategy and reinforced a referral system to delegate care appropriately. Technical assistance also shifted attention to human resources. During the second phase of SMI, a human resources diagnosis to evaluate workforce issues in hospital settings is underway.

Regarding supply procurement, SMI in Chiapas focused on implementing input registries and stock monitoring systems. These efforts have led to increased drug and equipment availability as proven in measurements following the first operation. According to high-level KIs and health workers, these efforts have also led to improved quality of services, patient satisfaction, and demand generation.

SMI revamping of health information systems allowed for generation of information on a far greater scale than before. Monitoring systems employed ranged from dashboards, progress reports, and follow-up surveys that tracked progress of maternal and child health indicators and determined disbursement on a national level.

Ministry of health representatives in Chiapas routinely use SMI data at meetings to inform decisions, review progress, and prioritize problematic areas. Also through SMI, per local and external sources, health information systems have been strengthened, including improvements to recordkeeping practices, as a side effect of quality improvement, which ensured increased coverage, efficiency, and quality of care at the ground level. For example, clinicians have cited how tracking measurements has raised awareness and improved standards for postpartum care in hospitals.

*“The main changes have been in postpartum monitoring, this includes vital signs, uterine massages, and hemorrhaging to prevent complications and hysterectomy. We even implemented a sheet to have supporting evidence from nurses or the physicians that performs the first massage, they have to record what they do. The three-hour postpartum monitoring, we know we have to provide constant monitoring to guarantee that the woman leaves in good condition.”* – *Doctor*

Other examples include using SMI data to create supply and training checklists in Belize, budget assessments for equipment in Guatemala, and cold chain protocol adjustments in Mexico. This new environment reflects a knowledge-sharing model.

### Theme 4: the evidence-based practice promoted policy dialogue and scale-up of interventions [tenets 2, 4]

From design to implementation, KI and FGD interviewees have cited the use of evidence as fundamental in shaping interventions and decision-making processes. Original SMI strategies used a multiplicity of assessments and extensive literature review, namely country-specific evidence packages by IDB, such as barrier studies, cost-effectiveness studies, and network analyses; a potential health gains analysis by the Institute for Health Metrics and Evaluation; censuses and health facility data sources; and mortality information systems. This early use of evidence set a precedent for later policy dialogue and scale-up of interventions.

On the policy level, data gathered from routine and external monitoring systems led to results-based actions, including forming best practices and quality improvement, adjusting existing protocols, and creating new guidelines. In Chiapas, policy changes included adherence to patient care guidelines and recordkeeping, and the creation of a quality of care unit and a logistical unit in the ministry of health.

*“We had data from the baseline throughout time. We got to our first operation and the scores that we got in the evaluation was not enough in order to approve it. And in that moment, we used all the information regarding what we wanted to achieve, what they would lack, what they would stop doing. And based on that, we generated new policies. Two of them are very strong which are the quality management unit and the logistic unit*.*”* – *ISECH*

In other SMI areas, some of the policy products of these evidence-based practices involve generating micronutrient standards for children in Honduras, improving the referral and counter-referral systems for all levels of the health network in El Salvador, improving community health worker skills in Guatemala, revising policies relating to child nutrition, namely oral rehydration solution and zinc in all SMI participating countries except Belize, and supporting the existing Essential Obstetric and Newborn Care system in all SMI participating countries.

In addition to policy adoptions, the evidence-based practice of SMI is evident in the scale-up of interventions to non-SMI regions. As discussed earlier, SMI quality improvement programs led jointly by MSH and ISECH in Chiapas have reportedly proven beneficial, spurring national stakeholders to reproduce these programs in other parts of Mexico. Similar scale-ups of SMI interventions are visible in other participating countries. In Panama, as migrant indigenous communities are receiving better health services in SMI areas, SMI is seeping through to other similar communities, while in Honduras, hospital support committees are being replicated in non-SMI facilities across the country.

## Discussion

We have shown that SMI, an ongoing health intervention in Mesoamérica, presents a promising potential for sustainability due to many features that fit within the DSF and subsequent application of lessons learned by the global community. Promising features include a flexible design that aligns with national health needs; partnerships and prioritization of health care; health systems strengthening in terms of quality improvements, capacity building, and health information systems; and policy products and scale-up of interventions. We also recognize the threats to sustainability, such as a culture of prioritizing health care that may be transient, the likely dissipation of reputational risk and donor-government financial partnerships, and frequent personnel turnover, which counter sustainability.

Chambers et al. proposed the DSF, which recognizes that traditional concepts of sustainability used in translational research are challenging to apply to real-world programs, particularly in low-resource settings [38, 39]. Instead of a static model of intervention development, DSF highlights an adaptive type in which change exists and should be embraced with constant improvement, evaluation, and learning; increasing rather than diminishing outcomes over time; context awareness; and partnership engagement.

### “Not optimized prior to implementation” (DSF tenet 1)

Per Chambers, the traditional linear process, used in the pharmaceutical industry, for example, suggests a drug be fully optimized during efficacy trials before implementation in the actual market. DSF notes that for a public health intervention to be sustainable, optimization occurs constantly over time, well after a program is implemented. The design of the intervention should be adaptive, receptive to adjusting, refining, and optimizing. SMI’s design remains flexible due its overarching TOC that factors in the evolving conceptual questions of the project. The adaptable TOC constantly optimizes SMI by its fluidity to add evidence, integrate changing practices, and gather inputs from contextual, knowledge, and policy assessments. The TOC is a multidimensional, comprehensive model rather than a linear one.

### “Ongoing stakeholder involvement” (DSF tenet 7)

According to the DSF, stakeholders should be involved throughout the planning, implementation, and adaptation phases, as this leads to an enhanced match between intervention and context and mitigating barriers toward long-term success. Stakeholders should also demonstrate partnerships with other players involved and not solely contribute to their individual domain. As illustrated, SMI stakeholders extended beyond their specified roles, with the ability to work together on all appropriate levels and stages of the project. During the planning stage, external donors and in-country representatives actively engaged with each other to create the Master Plans. Later on, as part of the adaptive phase for Mexico to improve after not reaching target indicators, partnership between the federal and state departments intensified, with heightened synergy, communication, and ownership. SMI further expanded on this partnership with the development of the regional approach and RBA model. These models enhanced a match between intervention and context through reputational risk, inter-country learning, accountability, and ownership. In particular, SMI demonstrated that countries reportedly can afford SMI costs, which represent a small fraction of their health expenditure, and that they have started to expand SMI activities to non-SMI regions.

### “Continually improves; ongoing feedback; organizational learning” (DSF tenets 2, 3, 6)

A mainstay of sustainability in the DSF is embracing constant improvement, evaluation, and learning. For an intervention to continually improve and enable ongoing learning, resources for quality improvement and training and ways to enhance efficiency and streamlining must be evident. Based on KIs and a sample of health care providers, SMI has reportedly brought continuous improvement through a sector-wide approach and quality improvement initiatives, namely process improvements and management practices. In partnership with ISECH, external technical assistance guided and financed by SMI developed quality-improvement programs centered on family planning and obstetric and neonatal care. Management practices and protocols implemented improved supervision and training of health care workers, standardization of work processes, and guidelines on emergency clinical scenarios and vaccine quality assurance.

The evaluation component of sustainability in the DSF occurs through ongoing feedback. An intervention must have the means to track changes, with relevant measures of progress. Ideally, feedback should be a shared process through collaboration rather than a linear one. By providing access to information at all levels of the health system, SMI employed monitoring systems to track changes and measure progress. On a macro level, SMI uses measurement follow-up surveys by which external evaluators, donors, and country representatives collaboratively could follow the progress toward meeting maternal and child health indicators. On a micro level, SMI developed a stock monitoring system to increase coverage, efficiency, and quality of physical resources in hospitals and clinics.

Organizational learning emphasizes a problem-solving capacity as part of the intervention to adapt quickly to an ever-changing environment. SMI possesses a problem-solving capacity. In Chiapas, failure to meet SMI goals in the first operation of the initiative due to lack of commitment led SMI stakeholders to execute an emergency performance-improvement plan. As a solution, this adaptive strategy rolled out activities that were not affected by delays and scaled up monitoring systems and technical assistance.

### “Fit between program and setting; voltage drop not inevitable” (DSF tenets 5, 4)

Some of the potential legacies of SMI can be evaluated through the lens of the last two DSF tenets. Per the DSF, context awareness is a necessary component of sustainability. A strong fit should exist between intervention and implementation setting, with the ability to align, understand the contextual constructs, and integrate with the practice environment and population over time. SMI’s TOC aligned with country health care priorities. SMI indicators were derived from the health standards of each country, and the SMI long-term goal of reducing maternal and child mortality and morbidity was a shared objective of national health plans. Examples of context awareness and understanding of the population’s needs include quality-improvement programs focused on cultural sensitivity around family planning and obstetric and neonatal care, and engagement of midwives to address cultural sensitivity around pregnancy. In sum, SMI’s fit with the environmental context will likely lead to the lasting gains of aligning with national health plans and strengthening health systems.

The final DSF tenet states that voltage drop is not inevitable, which means that a sustainable intervention would be maintained or possibly increase over time. Such interventions do not necessarily yield lower benefits when moving beyond an ideal setting into the real world. Chambers describes this concept akin to developing a better version of software in the computer industry, with improvements in performance, enhanced benefits, and a wider application. For SMI, a culture of prioritized health care emerged that resulted in improved performance. Stronger partnerships, the regional approach, and the RBA model promoted a culture of collaboration, inclusiveness, engagement, reputational risk, inter-country learning, ownership, and accountability. Such positive attitudes have become the new norm, raising expectations in the health care sector of these countries. Ongoing policy dialogue and scale-up of SMI programs exemplify the enhanced benefits and wider application characteristic of a higher voltage, and thus favorable long-term returns of the initiative. Lasting legacies include disease-specific policy guidelines, reinforcing the Emergency Obstetric and Neonatal Care system, assimilating SMI interventions into national packages, and expanding SMI interventions to non-SMI areas. Policy products and scale-up represent some of the tangible, visible contributions of SMI for years to come.

### Threats to sustainability

While our findings suggest a promising chance of sustainability of SMI at this midway point, some threats to sustainability affecting the initiative must be addressed. First, per some KIs, the culture of prioritizing health care that has emerged in Chiapas may be transient, in the setting of failure to meet target indicators and the need to urgently work on ensuring SMI funds for the following two operations. This refers to the strengthened collaboration between SSA and ISECH, and the efforts that ISECH made to meet their targets. However, other interviewees do sense a new norm in the health care sector, one that is long-lasting, with SMI’s financial incentive representing a small fraction of national health expenditures. Second, with the loss of a formal SMI umbrella structure, the regional reputational risk and the donor-government financial partnerships might dissipate. Without SMI targets to achieve, and with the loss of routine monitoring and measuring, the peer pressure not to fail publicly, which has been an important factor driving the success of SMI, may lessen. However, SMI is not the only framework through which these countries act cohesively. It is hence possible that this reputational risk concept could persist through other frameworks already in place, such as COMISCA. Third, turnover of personnel and administrative structure over time can impact the alignment of SMI and national health plans. Training of new personnel needs to be reinforced. However, it is highly improbable that maternal and child health would not remain a priority in the region given its long history of high maternal and child mortality.

Opposed to these threats is the fact that SMI has the opportunity to instigate a long-lasting behavioral change in the participating countries. While we recognize assessing sustainability of a health program, its counter-threats, and human behavior is a complex issue, we offer the following theory about behavioral change as one, of many, possible explanations to forecast the post-donor period. Behavioral change can be most successful with positive reinforcement strategies as previously demonstrated [40, 41]. According to Millon & Everly [42], behavior patterns are adopted based on reward and punishment. Humans engage in a wide variety of behavior responses as an exploratory function. This helps them learn what behavior responses lead to reinforcement and which responses are ineffective or punishing. Consequently, the human behavior gets shaped into preferred patterns of behaving. The previous success of these patterns makes them high-priority response patterns. Based on this theory, it is reasonable to expect that the behaviors adopted during SMI will be maintained, as countries will be repeatedly exposed to the same strategies of advancing to the next operation of SMI and receiving a performance tranche when reaching their targets, or risk expulsion from the initiative in the opposite case. However, countries are getting more than these two reinforcements. Within the accountability framework of SMI, measurement surveys are used to indicate whether countries have reached their target indicators. This gives countries an added third opportunity of seeing the evidence of their hard work through improved health indicators among their poorest populations.

## Lessons learned for the global community

In light of sustainability of global health programs gaining more attention, this analysis offers a considerable impact to the field as a prospective midway process evaluation. In a systematic review of the sustainability of health programs, most study designs were found to be retrospective [2]. Sustainability should not be viewed as a latent concern [7] or sequential to implementation, but rather a parallel process [26]. This study demonstrates that early consideration of sustainability during the implementation phase is useful in predicting potential lasting legacies, as well as identifying weaknesses to address and reallocating funds appropriately before a program ends.

Equally important, a standardized approach by using a sustainability model, such as the DSF, should be emphasized. For example, a review of health interventions in sub-Saharan Africa noted that only four of 41 interventions utilized a sustainability framework for assessing long-term outcomes [32]. Likewise, limited examples of sustainability frameworks, such as the Child Survival Sustainability Assessment in Bangladesh and the Sustainability Analysis Process in Nepal, exist in Asia [29, 43]. These are regions of the world with populations similar to the one in our study that may likely benefit from replicating our approach.

In parallel, it is important to note that while the DSF covers many dimensions of sustainability and more than those covered by other frameworks, it has its own shortcomings. For instance, the DSF does not address financing directly. Global health initiatives are mostly, if not entirely, funded by external donors as recipient countries are those of low or middle income. Changing policies in these countries to ensure commitment after cessation of external funds is important but would be irrelevant if the finances were not constantly secured. The DSF can be improved through additional research to cover this dimension.

While the sustainability of a public health program is often defined by scale-up of interventions and policy dialogue [30, 44], novel funding methods, in conjunction with regional partnerships and a sector-wide approach, are a distinctive characteristic of the Salud Mesoamérica Initiative that should be highlighted for valuable application in other areas of the world. The innovative RBA principles of using performance indicators and conditional criteria for measurable results promote country-led investments, ownership, and accountability by the countries involved [22]. Several RBA projects already exist worldwide but are often country-specific such as in Rwanda, Haiti, or Cambodia [45–47] or with a narrower agenda, like Gavi, the Vaccine Alliance [48]. To our knowledge, SMI is one of the first RBA initiatives that initiates collaboration on a regional rather than country-specific level, as well as seeking broader sector-wide goals rather than more focused ones. SMI is an RBA program that sparks intercountry learning and peer pressure on a regional level, while developing quality improvements, building capacity, and revamping health information systems across the health system sector in each country. This unique combination – RBA with regional partnerships and a sector-wide approach – should be considered for replication in other regions globally.

## Limitations

This study has some limitations. The first is social desirability on the part of some KI, such as national and local stakeholders, who might have emphasized the positive aspects of SMI and omitted the negative ones, given that they are the direct recipients of external donor funding. However, findings from the first operation and success thus far of the initiative substantiate positive findings of this evaluation. Second, memory bias and frequent staff turnover may limit data collected from interviews, but this has been compensated by supplemental document review. Third, local-level analysis, such as the social network analysis and case study of implementation, was limited to Chiapas, Mexico, but KI and FGD interviews provided substantial data from other countries at the regional level. Fourth, for the sustainability framework used, DSF does not explicitly discuss financing, which is an important factor to consider when evaluating the sustainability of an intervention, but this is implicitly included in the DSF tenet regarding partnerships. Fifth, this is a midway evaluation. Sustainability must be reassessed after some time has elapsed following the cessation of external SMI funds.

## Conclusion

Most SMI aspects fit within the DSF, suggesting a promising likelihood that gains and changes introduced due to SMI will be sustainable after external funding ceases, with subsequent wider application of lessons learned by the global community. Promising aspects include an evolving design that aligns with national health needs; partnerships and a culture of prioritizing health care; strengthened health systems through quality improvements, capacity building, and revamping of health information systems; and policy dialogue and scale-up of effective interventions. However, concerns about the possibility that the culture of prioritizing health is transient, the likely dissipation of reputational risk and donor-government financial partnerships, and frequent personnel turnover must be addressed to attain sustainability. Future assessments will help confirm whether this potential for sustainability has been maintained.

## Additional files

Additional file 1: Methods. (DOCX 15 kb)

Additional file 2: Global, National, and Local key informant Topic Guide (Group 1). (DOCX 26 kb)

Additional file 3: Health Care Providers key informant Topic Guide (Group 2). (DOCX 16 kb)

Additional file 4: Focus Group Discussion Topic Guide. (DOCX 21 kb)

Additional file 5: Ethical Considerations. (DOC 87 kb)

## Abbreviations

COMISCA: Consejo de Ministros de Salud de Centroamerica
DSF: Dynamic Sustainability Framework
FGD: Focus group discussions
IDB: Inter-American Development Bank
ISECH: Instituto de Salud del Estado de Chiapas
KI: Key informants
KII: Key informant interviews
MSH: Management Sciences for Health
PARTNER: Program to Analyze, Record and Track Networks to Enhance Relationships
RBA: Results-based aid
SMI: Salud Mesoamérica Initiative
SSA: Secretaría de Salud of Mexico
TOC: Theory of change
