# Supplementary material for: Results-based aid with lasting effects: sustainability in the Salud Mesoamérica Initiative
Source: Global Health. 2018 Oct 16;14:97. doi: 10.1186/s12992-018-0418-x (PMC6192274; doi:10.1186/s12992-018-0418-x)
Supplement: Supplementary file 1 — Methods. (DOCX 15 kb) [file 12992_2018_418_MOESM1_ESM.docx]

From May to October 2016, qualitative data collection was conducted for the Salud Mesoamérica Initiative Process Evaluation. Qualitative methods consisted of document review (DR), key informant interviews (KII), focus group discussions (FGD), and partnership analysis. Interview questions sought to address the overarching criteria recommended by the Development Assistance Committee of the Organisation for Economic Co-operation and Development for evaluating developmental aid, which include sustainability along with relevance, effectiveness, efficiency, and impact. The overall design of the evaluation was conducted on a regional level, with a case study of implementation at the local level in Chiapas, Mexico. The evaluation received Institutional Review Board exemption as a non-human-subject research determination from the University of Washington, and verbal consent was obtained from all study participants prior to interviews and data collection.

## Document review (DR)

We reviewed the operational plans, initiative proposals, annual and quarterly reports, national health norms, master plans for specific health areas, local official reports and meetings, needs assessments reports, indicator and target descriptions, and monitoring and evaluation documents.

The document review was used to refine research questions, develop topic guides, identify potential stakeholders to interview, describe the initiative, triangulate interview findings, retrieve missing information unable to be provided by key informants, and assess the theory of change.

## Key informant interviews (KII)

***Topic guide development:*** We generated topic guides for KIIs based on the document review, stakeholder exchanges, and fact-finding missions. Topic guides concentrated on issues pertaining to SMI planning, design, implementation, theory of change, efficiency, and lessons learned, as well as stakeholders’ decisions, resource allocation, and effectiveness. KII topic guides were modified based on the specific key informant audience, such as donors, Inter-American Development Bank (IDB), Project Coordinating Unit in Chiapas, Management Sciences for Health (MSH), Secretaría de Salud of Mexico (SSA), Instituto de Salud del Estado de Chiapas (ISECH), jurisdiction leaders, and health care providers.

***Sample selection:*** We compiled a list of 113 key informants (KIs) based on the document review and stakeholder recommendations. To be eligible, KIs selected must have been involved in one or more of the following: design and funding of SMI in general; design, planning, and implementation of the SMI operation for Chiapas; or working at the ministry of health in an SMI country.

KIs fell into two main groups: decision-makers and programmatic actors. The decision-maker group was composed of SMI partner organizations, including SMI funders (global key informants), IDB, technical assistance partners, ministries of health from multiple SMI countries (national key informants), and the Chiapas ministry of health (local key informants). The programmatic actor group consisted of health care providers and managers of health care facilities in Chiapas.

***Data collection:*** Prior to interviews, participants received an email or introductory letter describing the purpose of the study and interview topics. Participants were informed that responses would remain anonymous and confidential. Interviews ranged in duration from 60 to 90 minutes, conducted in the KI’s native language of either English or Spanish with an interpreter if necessary, and primarily in an in-person format at the participant’s work locale or private space. Due to location issues, four interviews were conducted via Skype.

Researchers leading the study conducted the interviews. CEB and BH, professors at the University of Washington with PhD degrees in biological anthropology and psychology, respectively, directed interviews for the first group (Additional File 2), accompanied by a data analyst for notetaking. Eight researchers from El Colegio de la Frontera Sur in Chiapas, with bachelor’s degrees in social sciences and training on the relevant study tools and procedures, led interviews for the second group (Additional File 3).

## Focus group discussions (FGD)

***Topic guide development:*** To gain a regional-level perspective of the SMI design and implementation from countries’ perspectives, a topic guide was created for representatives of the Ministries of Health from SMI countries involved in SMI implementation in their relative countries (Additional File 4).

***Sample selection:*** Ministry of health representatives from Honduras, Guatemala, Costa Rica, Belize, El Salvador, and Panama were present at the Latin American and Caribbean Conference 2016, and concurrently served as the nine participants sampled for the FGD.

***Data collection:*** Prior to interviews, participants received an email describing the purpose of the study and interview topics. Participants were informed that responses would remain anonymous and confidential. Conducted by CEB in English with an interpreter and audio-recorded, the focus group interview lasted 90 minutes with nine participants.

## Social Network analysis

***Social network tool design:*** We used the PARTNER (Program to Analyze, Record and Track Networks to Enhance Relationships) tool, designed by the University of Colorado, to demonstrate how members are connected, assess the degree of collaboration and engagement among stakeholders over time, evaluate the level of trust in the initiative network, and understand SMI objectives and outcomes. KIs representing the organizational stakeholders of SMI were asked to respond to questions measuring their perception of partner organizations, and questions about SMI focused specifically on Chiapas. They also responded to multiple-choice questions regarding their views on SMI’s objective, its success, and the aspects of collaborative work that propel the success it has achieved so far.

***Sample selection:*** KIs representing the organization stakeholders of SMI were chosen, including donors, IDB, technical assistance partners, evaluation partners, ministry of health officials from departments at the federal, state, and jurisdictional levels, and the evaluation organizations.

***Data collection:*** A PARTNER questionnaire was administered in English and Spanish both on paper and online.

## Data Analysis

All interviews from KII and FGD were transcribed verbatim from the audio recordings, and if needed, translated to English. Next, four researchers with educational backgrounds in health metrics, public health program evaluation, or social sciences manually coded and analyzed interview content through recursive abstraction. This process involved four steps. First, we organized transcribed data by topic and respondent to form a matrix. Second, we formed code-like sentences from extracted topic-relevant comments. Third, we generated summary narratives from coded responses of stakeholders within each respondent group. Fourth, we structured themes, which covered a number of codes to answer evaluation questions. Fifth, themes pertaining to sustainability were then compared against the DSF framework tenets. To screen for data saturation in each of the different groups, data analysis was conducted in parallel to data collection.

Data collected through the PARTNER tool were analyzed for connections between respondents to visualize the centrality of each organization and the number of organizations it is connected to. This allowed us to verify whether any outliers existed in the social network.
