# Supplementary material for: Results-based aid with lasting effects: sustainability in the Salud Mesoamérica Initiative
Source: Global Health. 2018 Oct 16;14:97. doi: 10.1186/s12992-018-0418-x (PMC6192274; doi:10.1186/s12992-018-0418-x)
Supplement: Supplementary file 3 — Health Care Providers key informant Topic Guide (Group 2). (DOCX 16 kb) [file 12992_2018_418_MOESM3_ESM.docx]

**What is your current position?**

How long have you been in this position?

Can you describe your major tasks and responsibilities?

**Knowledge of SMI**

Have you heard of the Salud Mesoamerica Initiative?

If yes, can you describe it for us?

What is your involvement in SMI?

Does the Mesored or jurisdiction consult with you regularly about the needs of your community? When was the last time this happened?

**Implementation**

To what extent have the activities contained in SMI’s plan been implemented as planned (quality, quantity and terms)?

What are the factors that affect, positively or negatively, the implementation of activities?

Are individual facilities rewarded if they meet their goals, and if so, how?

What happens if they don't meet their goals?

**Communication/responsiveness**

Can you compare the responsiveness from ISECH/Jurisdiction toward this facility before and after SMI?

Can you compare the responsiveness of this facility toward the community before and after SMI?

**Management and governance**

Can you compare the norms and protocols in this facility before and after SMI? BY NORMS AND PROTOCOLS, WE MEAN ANY WRITTEN RULES OR DOCUMENTS THAT GOVERN THE WAYS YOU PRACTICE HEALTH CARE IN THIS FACILITY.

How have these changed, if at all?

Is this due to SMI?

Can you compare the management of this facility before and after SMI? BY MANAGEMENT, WE MEAN THE WAYS THINGS ARE COMMUNICATED AND ACTIVITIES ARE ORGANIZED IN THIS FACILITY.

How has management changed, if at all?

Is this due to SMI?

How is this facility supervised by the jurisdiction?

Can you compare the supervision of this facility before and after SMI?

Can you compare the decision making process at this facility before and after SMI?

How has decision making changed, if at all?

Is this due to SMI?

Can you compare the operation and processes of this facility before and after SMI?

What are the changes, if any?

Is this due to SMI?

Can you compare the processes for Maternal and Child Health service provision before and after SMI?

Any changes for vaccine delivery/family planning methods/antenatal care & delivery care provision?

**Service provision**

What are the new procedures, activities and services introduced because of SMI?

Are these changes aligned with the community’s needs?

Can you speak specifically to family planning, ANC, delivery care, PNC, nutritional supplements, and vaccination?

What is the process in place for pregnant women with complicated pregnancies?

Has there been any change in health facility practices based on the preferences of different groups in the community?

What can be changed at this facility to satisfy the needs and preferences of different groups in the community?

How has the demand for services changed because of the newly introduced procedures, activities and services?

How is the demand affected by what services are available?

Do you feel that the users are satisfied with the new procedures, activities and services?

What affects their satisfaction?

What measures have been taken to improve the quality of care in this facility?

**Human resources**

Can you compare the human resources in terms of quality and quantity in this facility before and after SMI?

Have the personnel here received the training they need to perform the tasks necessary to do their jobs?

How have training activities changed as a result of SMI?

What are the effects of the trainings they received on record-keeping, family planning, child health care, antenatal and postnatal care, delivery services, management of obstetric emergencies…?

Task shifting

Has there been a change in tasks and responsibilities as a result of SMI?

Has there been recruitment of additional employees as a result of SMI?

What are the positions for which you recruited as a result of SMI?

What are their activities?

Are their activities conducted only in the facility?

What activities do they conduct outside of the facility?

Are there employees at this facility who provide training to other staff members, including training care providers?

How are recipients of training tested for new knowledge?

Do these employees who provide training also provide care?

Do these employees who provide training undergo continued education or training as well?

Is any of the training and continued education supported by SMI?

How have the midwives been integrated into this new environment due to SMI?

What is their role in the process of pregnancy and delivery care?

What are the factors of failure and success in engaging them in that process?

How have the changes affected health workers?

Did you lose or gain health workers due to these changes?

How do you incentivize employees to stay at this facility?

Do you recruit employees from within your community?

How do health workers perceive the changes in tasks, responsibilities, technologies, and services?

Has their performance changed due to these changes?

**Logistics and supply**

Have new equipment and tools been introduced to your health facility due to SMI?

How has the process of integrating new equipment and tools gone?

Do the personnel have the right training to use the newly introduced equipment and tools?

Have they been able to adapt to the new environment created by the introduction of new equipment and tools?

In your opinion, do the health care providers appreciate the newly introduced equipment and tools?

Has there been a change in the management of medical products as a result of SMI?

Has the availability of different medical products changed as a result of SMI?

Can you speak specifically to essential medicines, vaccines, the cold chain, family planning products, and lab tests? Are these more readily accessible for use?

Has the change in supply of different medical products affected the demand for services at your facility?

How is the demand affected by what products are available?

How has the resulting change in demand affecting your supply of medical products in return?

**Information Systems**

Can you compare the way you collect, manage, and use information at this facility before and after SMI?

What are the changes introduced to the health information system?

Specifically, for medical record keeping?

What is the effect of these changes on the service provision and the overall management of care at this facility?

What are the different uses of the health information collected at this facility?

How often are the collected data discussed between employees at your facility?

What is the purpose of these discussions?

How are patients tracked over time and followed across facilities?

Is there a system to share patient information between facilities?

How do you track pregnant women?

Does the facility search for pregnant women?

Is the search recorded?

If a woman stops coming for appointments, is that recorded?

What is done if a pregnant woman stops coming for appointments?
