# Supplementary material for: Results-based aid with lasting effects: sustainability in the Salud Mesoamérica Initiative
Source: Global Health. 2018 Oct 16;14:97. doi: 10.1186/s12992-018-0418-x (PMC6192274; doi:10.1186/s12992-018-0418-x)
Supplement: Supplementary file 5 — Ethical Considerations. (DOC 87 kb) [file 12992_2018_418_MOESM5_ESM.doc]

March 28, 2016

PI:	Dr. Ali Mokdad,

Global Health

CC:	Ms. Casey Evans

Re:	Human Subjects Application #51649,

“Salud Mesoamerica Initiative Process Evaluation”

Dear Dr. Mokdad,

The Human Subjects Division received the above-named Exempt Determination Request on March 23, 2016. This application has been reviewed by Subcommittee EJ. As the application describes, this activity involves assessment of Salud Mesoamerica Initiative (SMI) processes in order to explain the degree to which SMI’s intermediary and final outputs, outcomes, and impacts have been achieved. You have confirmed that:

1. You will be gathering information through interviews and focus groups about the design, implementation, results, efficiency, sustainability, and partnership of SMI.

2. Data collected for this project is intended to be used for SMI’s own internal operational monitoring and program improvement purposes. Results of this program evaluation will be shared with Inter-American Development Bank, Ministry of Health Chiapas, and others.

Based on this information and the definition of “research” under 45 CFR 46.102(d), the UW Human Subjects Division has determined that this activity does not meet the federal definition of “research.” This determination means that the activity is not subject to 45 CFR 46 and does not require review by the IRB. Please keep a copy of this letter for your records.

If you have further questions or concerns, feel free to contact me.

Best regards,

Kristen Wittmann

Human Subjects Review Administrator

Committee J

(206) 221-2093

kmw89@uw.edu
